# Membrane-Based Technologies for Water and Energy Sustainability

**DOI:** 10.3390/membranes11110807

**Published:** 2021-10-23

**Authors:** Xue Jin, Xin Liu

**Affiliations:** 1 School of Chemical, Biological and Environmnetnal Engineering, Oregon State University, Corvallis, OR 97331, USA; 2 School of Environmental Science and Engineering, Southern University of Science and Technology, Shenzhen 518055, China; liux5@sustech.edu.cn

In finalizing this Special Issue, “Membrane-based Technologies for Water and Energy Sustainability”, we would like to express our sincere appreciation to the authors, reviewers, and publisher for their outstanding work. Water and energy are two fundamental building blocks of society and the economy. However, water shortage is a global problem. The World Health Organization predicts that by the mid-twenty-first century, nearly two-thirds of the world’s present population will face severe freshwater shortages. Meanwhile, climate change and the increasing demand for global energy consumption expedite the development and innovation of renewable energy. Membrane technology plays an important role in the advancement of sustainable water and energy demands. Despite their promise, the successful industrial application of membrane technologies depends largely on developing high-performance membranes, optimizing operating conditions, improving reliable and robust system design, and validating economic-energy competitiveness. The membrane community all over the world is continuing to contribute to promoting membrane process efficiency towards the more sustainable and cost-effective generation of clean water and energy. 

This Special Issue consists of five research articles that disseminate the current knowledge of the above topics. We hope this collection will be useful for developing plans for future research topics on the application of membrane technology for water and energy sustainability. 

Wei et al. [1] investigated the influence of membrane permeability and structure on the performance of an osmotically driven membranes. Four commercial osmotic membranes were systematically characterized in terms of intrinsic separation parameters, as well as structure and surface properties. The results demonstrate that membranes with smaller structural parameters (S) and higher water/solute selectivity underwent lower internal concentration polarization (ICP) and exhibited higher efficiency. Under the condition with low ICP, membrane water permeability (A) had a dominant effect on water flux. In contrast, water flux became less dependent on the A value but was affected more by membrane structure under the condition with severe ICP, and the membrane exhibited lower FO efficiency.

Membrane bioreactors (MBRs) have great potential for wastewater treatment. Experiments carried by Gkotsis et al. [2] examined the effect of operating conditions on MBR fouling caused by filamentous microorganisms. Their results showed that a low temperature slightly increased the number of filaments, which, in turn, reduced membrane fouling. In addition, the low Food-to-Microorganisms (F/M) ratio prevented the significant growth of filamentous microorganisms, allowing the proteins and carbohydrates to be kept at very low values in the mixed liquor and thus contributing to fouling alleviation. 

Membrane-based separation technologies also play an important role in improving water quality to meet the requirements for various industrial applications. Membrane capacitive deionization (MCDI) could be a promising technology to deal with divalent ion-induced scaling. Zhang et al. [3] employed a process model coupled with ion exclusion effects to investigate the water softening performance in MCDI with a constant voltage mode; the trade-off between the simulated calcium ion selectivity and removal efficiency was observed under varied conditions. Their results showed that the specific energy consumption of MCDI is an order of magnitude less than the reported values of other water softening techniques. 

Xu and coworkers [4] focused on the reclamation of municipal wastewater using reverse electrodialysis (RED) for irrigation. The effects of operating parameters on water quality were investigated by implementing bench and pilot-scale experiments with a RED system containing ion-exchange membranes. The techno-economic feasibility of the RED treatment in water reclamation was assessed, and it was shown that it could be more cost-effective than a UF/RO hybrid system. In addition, the overall water recovery of a RED system was higher than that of the UF/RO system. 

In a study by Davydov et al. [5], they employed a micro-heterogeneous model to estimate the transport numbers of counter ions through various commercial ion-exchange membranes in a RED system. To select the best membrane pairs for a RED process, the characteristics of the ion-exchange membranes, such as the electrical conductivity and diffusion permeability, were calculated and compared with the experimental data. Their results suggested that the calculated values were highly dependent on the choice of ion-exchange membranes, and the discrepancy between the experimental results and theoretical data might be ascribed to the shadow effect of the spacer in the channels. 
